# Diagnostic Accuracy of Ex Vivo Confocal Laser Scanning Microscopy for Routine Detection of Cutaneous Squamous Cell Carcinoma and Actinic Keratoses

**DOI:** 10.3390/cancers18091458

**Published:** 2026-05-01

**Authors:** Viktor Schnabel, Conrad Hempel, Mirjana Ziemer, Jan C. Simon, Sonja Grunewald

**Affiliations:** Department of Dermatology, Venereology and Allergology, University Medical Center Leipzig (AöR), 04103 Leipzig, Germanysonja.grunewald@medizin.uni-leipzig.de (S.G.)

**Keywords:** ex vivo confocal microscopy, CLSM, squamous cell carcinoma, actinic keratosis

## Abstract

**Background and Objectives**: Invasive cutaneous squamous cell carcinoma (cSCC) and actinic keratosis (AK) are growing burdens on ageing societies and mainly arise from chronic sun exposure. Distinguishing between carcinoma and carcinoma in situ is often clinically challenging but essential for decision-making with respect to targeted therapy. Ex vivo confocal laser scanning microscopy (CLSM) allows histologic examination of native tissue using tissue reflection and nuclear fluorescence staining. The digital staining process almost perfectly mimics conventional haematoxylin and eosin (HE) staining. While the use of CLSM for basal cell carcinoma (BCC) diagnosis is well described, data on cSCC remain scarce. The aim of this study was to compare CLSM with conventional histology in diagnosing and distinguishing cSCC and AK in routine clinical practice. **Materials and Methods**: Between August 2022 and March 2024, 63 patients with clinically suspected invasive cSCC or AK were enrolled. Biopsy or excision specimens were examined by ex vivo confocal laser scanning microscopy (VivaScope^®^ 2500 M-G4) followed by acridine orange staining. All samples underwent subsequent routine histopathology, which served as the reference. Two dermatologists independently compared the CLSM findings with the histopathological diagnoses. **Results**: Eighty-one lesions suspected to be cSCC were assessed using CLSM. The diagnostic accuracy was high across specimen types: cSCC was correctly identified in 24/26 cases (92.3%), AK/Morbus Bowen in 16/17 cases (94.1%), cSCC filia (cutaneous squamous cell carcinoma metastases) in 3/3 cases (100%), and basal cell carcinoma in 9/9 cases (100%). The absence of a tumour was correctly recognized in 19/21 cases (90.5%). In five cases (6.2%), AK and early invasive cSCC could not be differentiated, reflecting the histopathological results. **Conclusions**: CLSM with digital HE staining is well suited for rapid cSCC and AK diagnosis and differentiation in clinical practice.

## Simple Summary

Skin cancers such as invasive cutaneous squamous cell carcinoma and precursor lesions such as actinic keratosis are increasingly common, mainly due to long-term sun exposure and ageing populations. Rapid and reliable recognition is important because treatment decisions depend on accurate diagnosis. The aim of our study was to evaluate whether ex vivo confocal laser scanning microscopy, a fast digital imaging technique that examines freshly removed tissue, can support routine diagnosis in daily clinical practice. We compared its performance with conventional histology in patients with suspected lesions. Our results demonstrate that this method identifies most tumours with high accuracy, exceeding 92%, and may facilitate rapid assessment directly after tissue removal. These findings suggest that digital microscopy could improve clinical workflows, support earlier therapeutic decisions, and encourage further research into rapid diagnostic technologies in dermatology.

## 1. Introduction

Invasive cutaneous squamous cell carcinoma (cSCC) and actinic keratosis (AK) represent two highly prevalent and closely related entities within the spectrum of ultraviolet-induced epithelial skin damage. Both conditions primarily arise from chronic sun exposure and are further promoted by risk factors such as advanced age and immunosuppression [1,2,3]. While AK is regarded as an in situ lesion with a variable potential for invasion, invasive cSCC constitutes a neoplasm associated with significant morbidity, and in advanced stages, disease-specific mortality [4,5]. In recent decades, the global incidence of both AK and cSCC has increased steadily, emphasizing the need for improved diagnostic and management strategies [6]. From a clinical perspective, the growing burden of keratinocytic neoplasms requires diagnostic approaches that combine accuracy with speed. Dermatological services increasingly face high patient volumes, making rapid tissue-based assessment particularly relevant.

The diagnosis of AK and invasive cSCC is currently based on clinical examination supported by histopathological assessment. However, clinical evaluation alone often lacks sufficient sensitivity and specificity, particularly when distinguishing between intraepithelial and early invasive carcinoma [7]. Although histopathology remains the diagnostic gold standard, it requires invasive tissue sampling and is limited by processing time [8]. These limitations have driven the development of adjunctive diagnostic tools that allow rapid and reliable tissue assessment while preserving specimens for conventional histology.

In this context, advanced imaging techniques such as dermoscopy, optical coherence tomography (OCT), and confocal laser scanning microscopy (CLSM) have become increasingly relevant in dermatological diagnostics [9,10,11,12,13]. Among these modalities, CLSM offers real-time, high-resolution visualization of skin architecture at the quasi-histological level. In vivo CLSM has been established as a non-invasive “optical biopsy” for various skin tumours; however, its clinical applicability is limited by a horizontal imaging orientation and a restricted penetration depth of approximately 250 µm [11,14].

These limitations can be largely overcome by ex vivo CLSM, which allows examination of freshly excised tissue specimens. By rotating the specimen, vertical sections comparable to conventional histological slides can be obtained, which enables detailed assessment of tumour architecture and surgical margins [15,16,17]. Ex vivo CLSM further permits fluorescent nuclear staining with agents such as acridine orange without compromising subsequent haematoxylin–eosin (HE) staining [18]. Recent technical advances have enabled digital HE-like staining through automated image processing, which has improved image interpretability and familiarity for dermatopathologists [19]. In addition, ex vivo confocal microscopy has been shown to significantly accelerate intraoperative margin assessment and reduce hospital stay in patients undergoing re-excision of skin tumours, highlighting its potential to improve clinical efficiency [20].

Previous studies have demonstrated the high diagnostic accuracy of ex vivo CLSM, particularly for the diagnosis of basal cell carcinoma [18,21,22], while evidence for invasive cSCC has been more limited [23,24]. Characteristic CLSM features of cSCC include clearly visible invasive growth patterns, nuclear pleomorphism, and keratinization; the keratinized areas show increased reflectance signals while tumour cells appear less eosinophilic than those in conventional HE sections [16]. These features suggest that ex vivo CLSM is fundamentally suitable for the diagnosis of invasive cSCC and its in situ lesions.

Beyond primary diagnosis, ex vivo CLSM has also been explored as a potential tool for intraoperative margin assessment and as an alternative to frozen-section analysis [25]. While this approach is well established for basal cell carcinoma, data for cSCC remain scarce. Available studies report heterogeneous sensitivities and specificities, and overall evidence is still limited, particularly for cutaneous cSCC [23,26]. This highlights a persistent diagnostic gap and the need for further systematic evaluation of ex vivo CLSM in keratinocytic neoplasms beyond basal cell carcinoma.

In addition to improving diagnostic accuracy, there is an increasing demand for technologies that enable faster decision-making and more efficient clinical workflows in dermatologic oncology. In this context, digital imaging techniques are gaining importance, particularly in settings with high patient turnover and limited resources. Rapid bedside or intraoperative assessment of excised tissue may help to optimize treatment planning and reduce delays associated with conventional histopathological processing.

Moreover, the ongoing digital transformation in pathology has opened new perspectives for integrating imaging modalities such as CLSM into routine diagnostics. The ability to generate and store high-resolution digital datasets not only facilitates interdisciplinary communication but also enables remote consultation and potential integration with artificial intelligence-based image analysis tools. These developments highlight the growing relevance of CLSM as part of a broader movement toward digital and precision-based dermatopathology.

Therefore, the aim of the present study was to evaluate the diagnostic performance of ex vivo CLSM with digital HE staining for the recognition and differentiation of cSCC and AK in routine clinical specimens and to directly compare this method with conventional histopathology.

## 2. Materials and Methods

This prospective study was approved by the local ethics committee of the Medical Faculty of the University of Leipzig (approval number O93/18-ek). Between August 2022 and March 2024, 63 patients with clinically suspected invasive cSCC or AK were consecutively enrolled, after providing informed consent. Eligibility criteria included age ≥18 years and a clinical diagnosis of cSCC or AK that required biopsy or excision in the dermatosurgical unit of the Department of Dermatology, Venereology and Allergology, University Hospital Leipzig. Inclusion criteria therefore comprised all consecutively enrolled adult patients with clinically suspected keratinocytic neoplasms. Exclusion criteria included lack of informed consent. For diagnostic evaluation, confocal images were independently compared with conventional histopathological sections by two experienced dermatopathologists.

### 2.1. Ex Vivo Confocal Laser Scanning Microscopy (CLSM)

**Figure 1:**
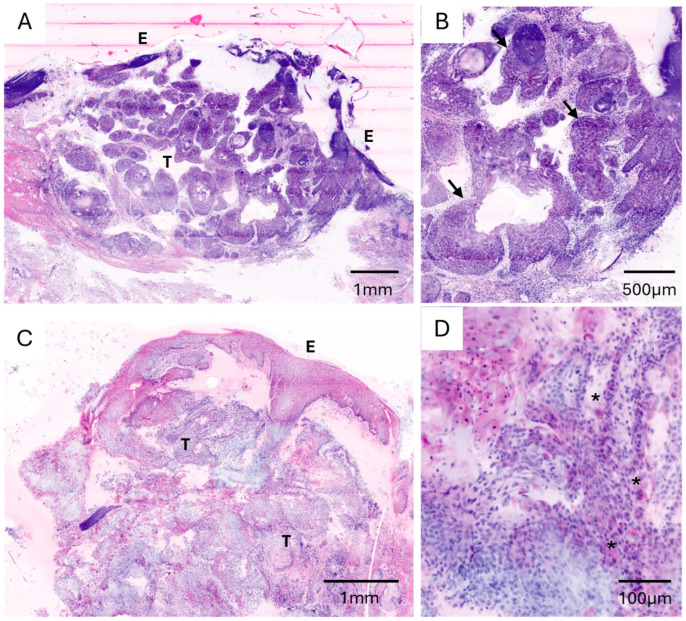
Ex vivo confocal laser scanning microscopy (CLSM) images of two different punch biopsies of invasive cutaneous squamous cell carcinoma (cSCC) (**A**–**D**). (**A**,**C**) Low-magnification overview demonstrating infiltrative tumour growth (T) originating from the epidermis (E). (**B**) Higher magnification showing infiltrating tumour cell nests (arrows) and areas of keratinization. (**D**) High-magnification image revealing numerous atypical cells with marked nuclear pleomorphism (asterisk).

Ex vivo imaging was performed using the VivaScope^®^ 2500M-G4 system (Lucid Inc., Rochester, NY, USA; distributed by MAVIG Germany GmbH) (Figure 1). This device is equipped with two laser sources operating at wavelengths of 488 nm (fluorescence mode) and 635 nm (reflectance mode). The tissue samples were examined using reflectance mode (RM), fluorescence mode (FM), and overlay mode (OM), which enabled simultaneous visualization of both signals. In addition, this system digitally simulates a haematoxylin–eosin (HE) staining, as previously described by Gareau et al. [19]. In accordance with the manufacturer’s specifications, the maximum scan field covers 24 × 32 mm, with an imaging depth of up to 200 µm, depending on tissue characteristics. Continuous magnification of up to 550× can be achieved.

### 2.2. CLSM Procedure and Conventional Histopathological Examination

Immediately after surgical biopsy or excision, tissue specimens were placed in 0.9% sodium chloride solution to prevent desiccation and were promptly processed for CLSM examination. All the samples were stained with acridine orange (0.6 mM; Sigma-Aldrich, Merck KGaA, Darmstadt, Germany) for 30 s, after which they were rinsed with phosphate-buffered saline (PBS) (Sigma-Aldrich, Merck KGaA, Darmstadt, Germany) for an additional 30 s to remove excess dye. For optimal image acquisition, a tissue-flattening technique using a system of magnets and sponges, as described by Pérez-Anker et al. [27,28], was applied. The prepared samples were then positioned on the CLSM stage. Prior to image acquisition, manual adjustments were performed to optimize the imaging depth and to balance the reflectance and fluorescence signals. A macroscopic overview image was used to define the scanning area and to guide navigation across the sample.

Following CLSM imaging, all the specimens were fixed in 4% buffered formalin, embedded in paraffin, sectioned, and stained with haematoxylin and eosin for routine histopathological evaluation. The histological slides were assessed independently and in a blinded manner by an experienced dermatopathologist. The histopathological findings were subsequently compared with the corresponding CLSM results. Diagnostic performance was assessed by calculating overall diagnostic accuracy using conventional histopathology as the reference standard. Given the categorical structure of the dataset, additional parameters such as sensitivity and specificity could not be comprehensively calculated for all diagnostic groups. Diagnostic accuracy was calculated as the proportion of correctly classified cases. Corresponding 95% confidence intervals were calculated using the Wilson method.

## 3. Results

Eighty-one clinical specimens from 63 patients suspected to have invasive cSCC or AK were included in the analysis and were evaluated using CLSM with digital HE staining. The specimen types included 14 excisions, 45 punch biopsies, and 22 shave biopsies.

The appearance of the cSCC cells in digitally simulated CLSM images was similar to that in conventional HE sections. However, tumour cells were less eosinophilic, and keratinization results in increased reflection. In contrast, nuclear pleomorphism appeared more pronounced in CLSM images. Ulceration, depth of invasion and grade of differentiation (highly differentiated tumours, G1) were easy to determine using ex vivo CLSM (Figure 1).

**Figure 2:**
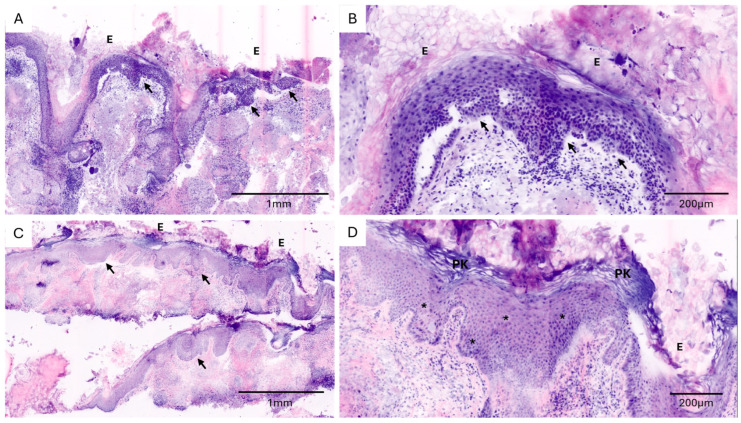
Ex vivo confocal laser scanning microscopy (CLSM) images of in situ keratinocytic lesions obtained from different specimen types ((**A**,**B**): punch biopsy of actinic keratosis; (**C**,**D**): shave biopsy of Bowen’s disease). (**A**,**C**) Low-magnification overview showing irregular epidermal architecture with focal acanthosis and dysplasia (arrows). (**B**) Higher magnification demonstrating atypical keratinocytes and disordered epidermal structure (arrows). (**D**) High-magnification image revealing cytological atypia with enlarged nuclei (asterisk) and focal parakeratosis (PK). E, epidermis.

**Figure 3:**
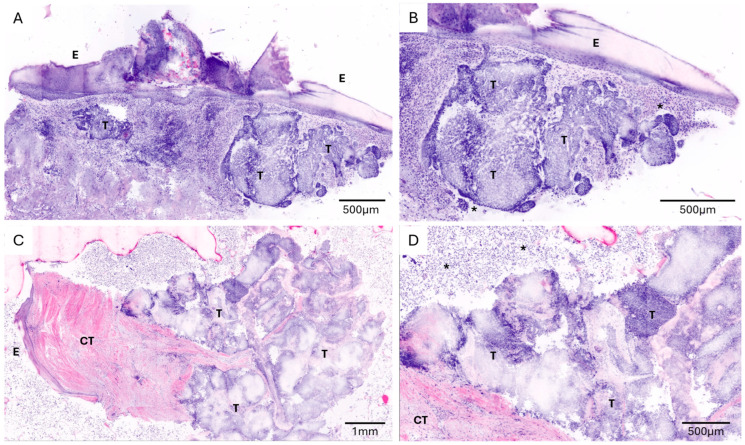
Ex vivo confocal laser scanning microscopy (CLSM) images of additional tumour entities. (**A**,**B**) Basal cell carcinoma (BCC); (**C**,**D**) cutaneous squamous cell carcinoma (cSCC) metastasis. (**A**) Low-magnification overview showing basophilic-appearing tumour islands (T) within the dermis. (**B**) Higher magnification demonstrating characteristic basophilic tumour nests with peripheral palisading (T) and peritumoural retraction artefact (asterisk). (**C**) Low-magnification overview of a cSCC metastasis showing deeply located tumour nests (T) within connective tissue (CT) without clear capsule formation. (**D**) High-magnification image revealing atypical epithelial cells with nuclear pleomorphism and a pronounced accompanying lymphocytic infiltrate (asterisk). E, epidermis; T, tumour; CT, connective tissue.

Overall, compared with conventional histopathological assessment, CLSM demonstrated high diagnostic accuracy across all specimen categories (Table 1). The diagnostic accuracy for invasive cutaneous squamous cell carcinoma (cSCC, G1) was 92.3% (95% CI: 74.9–99.1) (Figure 1). For in situ carcinoma (actinic keratosis/Morbus Bowen), the diagnostic accuracy was 94.1% (95% CI: 71.3–99.9) (Figure 2). Diagnostic accuracy was 100% for both cSCC metastases (95% CI: 29.2–100) and basal cell carcinoma (95% CI: 66.4–100) (Figure 3).

**Figure 4:**
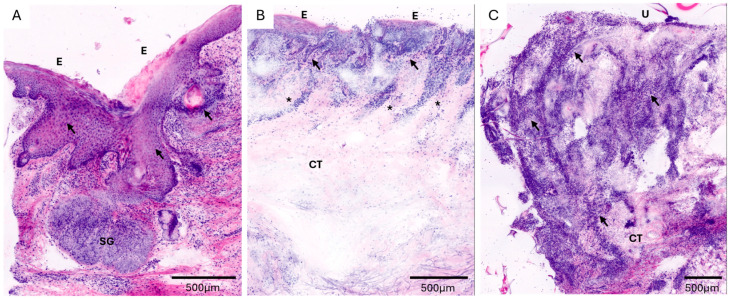
Ex vivo confocal laser scanning microscopy (CLSM) images of diagnostically challenging cases with discordant findings compared to histopathology. (**A**) Case initially interpreted as actinic keratosis in CLSM, showing acanthosis and polymorphic keratinocytes (arrows). Final histopathology after deeper sectioning revealed invasive growth consistent with early cSCC (false negative). (**B**) Case suspected as invasive cSCC in CLSM due to proliferating epithelial structures extending from the epidermis into the superficial dermis (arrows), with prominent lymphocytic infiltrates (asterisk). Final histopathology demonstrated seborrheic keratosis (false positive). (**C**) Punch biopsy from the scalp (capillitium) with marked ulceration. CLSM suggested invasive cSCC due to atypical cells (arrows) extending into deeper dermal layers; however, interpretation was limited by extensive inflammatory infiltrates. Final histopathology revealed erosive pustular dermatosis of the scalp (false positive). E, epidermis; SG, sebaceous gland; CT, connective tissue; U, ulceration.

In specimens without evidence of neoplasia on conventional histopathology, CLSM correctly revealed the absence of tumours in 19 of 21 cases, corresponding to a diagnostic accuracy of 90.5% (95% CI: 69.6–98.8). In five cases (6.2%), differentiation between AK and early invasive cSCC remained inconclusive on CLSM evaluation. However, two of these cases were also considered diagnostically ambiguous on conventional histopathological examination (Table 1). In a small number of cases, discrepancies between CLSM and histopathology were observed. These included both false negative and false positive findings (Figure 4). False negative cases were mainly associated with subtle or early invasive growth patterns that were difficult to detect, particularly in specimens with limited tissue depth. False positive findings were rare but may be explained by reactive or inflammatory changes mimicking tumour morphology in CLSM. In addition, diagnostically ambiguous cases were primarily observed in the differentiation between actinic keratosis and early invasive cSCC, reflecting the biological continuum of these entities.

Overall, these findings indicate that CLSM provides robust morphological information in the majority of cases, while diagnostic limitations are primarily confined to borderline lesions and technically challenging specimens. Importantly, the observed discrepancies were not random but could be attributed to identifiable factors, such as limited tissue depth or subtle histopathological transitions, which supports the interpretability and clinical reliability of CLSM findings.

Excision specimens and punch biopsies generally allowed reliable evaluation with ex vivo CLSM because of sufficient tissue depth and preserved vertical architecture. In contrast, sometimes shave excisions resulted in diagnostically uncertain findings, which was mainly attributable to limited tissue depth and difficulties with the correct placement of the sample during laser scanning. In such cases, we found it helpful to scan the bottom of the shave specimens first to obtain an image of the complete basal margin.

## 4. Discussion

In this study, CLSM with digitally simulated HE staining demonstrated high diagnostic accuracy for the evaluation of keratinocytic neoplasms in a routine clinical setting. Highly differentiated invasive cSCC and related in situ carcinomas were reliably identified, supporting the role of ex vivo CLSM as a rapid and robust adjunct to conventional histopathology in real-world clinical workflows, including outpatient surgical settings.

The morphological features of cSCC observed by ex vivo CLSM were consistent with previously described characteristic patterns [21], including invasive growth, nuclear pleomorphism, and keratinization. Keratinized areas showed increased reflectance signals, which facilitated tumour recognition.

Invasive epithelial tumours and in situ carcinomas such as AK were recognized with high reliability, with diagnostic accuracy exceeding 92%. However, the differentiation between actinic keratosis and early invasive cSCC remains a relevant diagnostic limitation of CLSM. This challenge reflects the biological continuum between intraepidermal dysplasia and early dermal invasion, which may present with only subtle morphological differences. In particular, the reliable detection of minimal invasion is inherently limited in CLSM, especially in superficially scanned sections. Notably, even conventional histopathology yielded inconclusive findings in a subset of these cases, underlining the intrinsic difficulty of this distinction. Therefore, CLSM findings should always be interpreted in conjunction with the clinical context, and histopathological confirmation remains essential in ambiguous cases.

Compared with earlier reports focusing primarily on basal cell carcinoma [18,21,22], our findings extend the evidence base for CLSM in keratinocytic tumours. The high concordance with histopathology suggests that digitally stained CLSM images provide sufficient morphological detail to support clinically meaningful decisions.

Specimen type had a relevant impact on diagnostic confidence. Punch biopsies and excision specimens were superior to shave biopsies, as they provided sufficient tissue depth and allowed more reliable assessment of the epidermal–dermal junction. In contrast, shave biopsies sometimes resulted in diagnostic uncertainty due to limited depth and truncated bases. From a practical perspective, careful specimen handling and preparation are crucial for optimal CLSM evaluation. Ensuring sufficient tissue depth and correct specimen orientation can significantly improve image quality and diagnostic interpretability. When feasible, punch biopsies or excisional specimens including an adequate dermal component should be preferred over superficial shave biopsies, particularly when invasive growth is clinically suspected. In cases where shave biopsies are used, scanning the basal aspect of the specimen may improve assessment of the tumour margin. These findings underscore the importance of appropriate specimen selection and preparation when ex vivo CLSM is used for the evaluation of keratinocytic lesions.

Beyond diagnostic concordance, our findings highlight the potential of CLSM to streamline interdisciplinary workflows between dermatologic surgery and dermatopathology. The rapid availability of quasi-histological images may help clinicians assess tumour behaviour at an earlier stage and adapt surgical strategies when clinically indicated. This is particularly relevant in outpatient settings, where treatment decisions often need to be made within limited timeframes. Moreover, the digital nature of CLSM datasets allows storage, re-evaluation, and remote consultation, which may contribute to improved quality assurance and training.

Recent evidence suggests that ex vivo CLSM may extend beyond qualitative diagnosis by enabling accurate measurement of tumour thickness, a key prognostic parameter in cSCC, with high concordance to histopathology, thereby supporting rapid intraoperative risk stratification [29].

In this context, CLSM may also contribute to improving diagnostic efficiency and reducing turnaround times in dermatologic practice. The immediate availability of quasi-histological images has the potential to support more timely clinical decision-making, particularly in outpatient settings where rapid therapeutic planning is essential. Furthermore, the integration of CLSM into routine workflows may facilitate closer collaboration between dermatologists and dermatopathologists, enhancing diagnostic consistency and reducing interobserver variability. An additional important aspect is the learning curve associated with CLSM interpretation. Dermatologists accustomed to conventional histopathology may require dedicated training to become familiar with CLSM-specific image characteristics and contrast patterns. This factor should be considered when implementing CLSM in routine clinical practice.

From a broader perspective, CLSM represents an important step toward the implementation of digital pathology solutions in dermatology. The combination of high-resolution imaging, digital storage, and emerging analytical tools may enable more standardized and reproducible diagnostic pathways in the future.

The main limitations of this study include the moderate sample size and the predominance of highly differentiated (G1) tumours, which may restrict extrapolation to more aggressive variants. In particular, well-differentiated tumours tend to preserve more distinct architectural features, which may facilitate interpretation in CLSM. In contrast, moderately or poorly differentiated tumours often exhibit less organized structures and more pronounced cellular atypia, potentially reducing diagnostic confidence. Therefore, the generalizability of our findings to less differentiated tumour subtypes remains limited and should be addressed in future studies including more histologically diverse cohorts. Given the moderate sample size and the exploratory nature of this analysis, the present study should be considered a pilot study. While the results are promising, further validation in larger and more diverse cohorts is required. Furthermore, this was a single-centre study, which may limit the generalizability of the findings. Validation in larger, multi-centre cohorts is required before broader clinical application.

The statistical analysis was primarily descriptive, and a comprehensive calculation of additional performance measures such as sensitivity and specificity was not feasible due to the structure of the dataset and the limited sample size. This should be considered when interpreting the results.

Future multi-centre studies with standardized evaluation criteria are warranted to confirm these findings and to explore the role of automated image analysis.

## 5. Conclusions

Overall, ex vivo CLSM with digital simulated HE staining enables rapid, histology-like visualization without tissue loss and preserves specimens for standard histopathologic processing and evaluation. While this method is highly reliable for highly differentiated invasive epithelial tumours, diagnostic limitations may persist in very early minimally invasive lesions, which emphasizes that CLSM should be regarded as a complementary technique rather than a replacement for histopathology.

Finally, the integration of artificial intelligence and machine learning-based image analysis represents a promising future direction for ex vivo CLSM [30]. Automated evaluation of large image datasets may facilitate the detection of subtle morphological patterns, improve diagnostic reproducibility, and support faster clinical decision-making. In routine practice, CLSM has the potential to contribute to more individualized diagnostic pathways by integrating imaging findings, clinical assessment, and histopathology into a structured and stepwise workflow. Future developments are expected to include standardized interpretation criteria, automated analysis tools, and integration into digital pathology platforms, which may enhance interobserver consistency and enable multi-centre collaboration. Nevertheless, careful consideration of training requirements, workflow integration, and cost-effectiveness will be essential to ensure sustainable clinical implementation. Larger prospective studies including diverse tumour subtypes are needed to validate these observations and to better define the clinical scenarios in which CLSM provides the greatest benefit. Taken together, our findings support ex vivo CLSM as a clinically meaningful complementary technique that enhances diagnostic efficiency while preserving the central role of conventional histopathology, highlighting its potential contribution to future precision-oriented dermatologic oncology.

## Figures and Tables

**Table 1:** Diagnostic performance of ex vivo confocal laser scanning microscopy (CLSM) for routine clinical specimens (*n* = 81), including diagnostic accuracy by histopathological diagnosis.

Histopathological Diagnosis	Correct CLSM Diagnosis/Total Cases	Diagnostic Accuracy (%)	Remarks
Invasive cutaneous squamous cell carcinoma (cSCC), G1	24/26	92.3	1 borderline case (histopathology inconclusive), 1 seborrheic keratosis
In situ carcinoma such as actinic keratosis/Morbus Bowen	16/17	94.1	1 borderline case (histopathology inconclusive)
cSCC metastasis	3/3	100	—
Basal cell carcinoma	9/9	100	—
No tumour	19/21	90.5	—
Indeterminate: in situ vs. early invasive cSCC	5	6.2	2 cases also inconclusive on histopathology

## Data Availability

The original contributions presented in this study are included in the article. Further inquiries can be directed to the corresponding author.
